# CjejuniTyper: integrated *in silico* typing of *Campylobacter jejuni* capsule and lipooligosaccharide loci with GBS-associated genomic profiling

**DOI:** 10.3389/fmicb.2026.1943123

**Published:** 2026-10-19

**Authors:** Dragan Jurić, Ivana Ferenčak, Ana Gverić Grginić, Ljiljana Žmak, Irena Tabain

**Affiliations:** 1 Croatian Institute of Public Health, Zagreb, Croatia; 2 School of Medicine, University of Zagreb, Zagreb, Croatia

**Keywords:** *Campylobacter jejuni*, capsular polysaccharide, ganglioside mimicry, Guillain-Barré syndrome (GBS), in silico typing, Penner serotyping

## Abstract

*Campylobacter jejuni* (*C. jejuni*) is the leading bacterial cause of gastroenteritis worldwide and the most common antecedent pathogen linked with Guillain–Barré syndrome (GBS), an acute autoimmune neuropathy. The risk of GBS development is associated with the structure of the bacterial lipooligosaccharide (LOS), especially sialylated LOS classes that mimic human peripheral nerve gangliosides, as well as with the Penner heat-stable (HS) capsular polysaccharide (CPS) serotype. Accurate simultaneous determination of LOS class and CPS type is important for surveillance of GBS-associated *C. jejuni* lineages but currently requires culture-based serology or separate molecular assays. CjejuniTyper is an automated bioinformatic workflow that performs Penner CPS complex prediction, LOS class assignment, *Cst-II* allele calling, putative ganglioside-mimic prediction, virulence-factor detection, and antimicrobial-resistance profiling from genome assemblies. Penner prediction uses marker-gene searches, full-locus alignment, and subtype-resolution rules, whereas LOS classes are assigned using a BLAST-based scoring and confidence system. The workflow was evaluated using 617 assemblies with previously assigned LOS classes, 96 *C. jejuni* assemblies with Penner reference labels, and 113 retained genomes comprising 89 isolates previously reported from GBS patients and 24 enteritis controls. LOS typing correctly classified 595 of 617 assemblies, corresponding to 96.4% accuracy, Cohen’s kappa of 0.958 and a Matthews correlation coefficient of 0.958. Penner typing correctly classified 93 of 96 assemblies, corresponding to 96.9% accuracy, Cohen’s kappa of 0.961, and a Matthews correlation coefficient of 0.962. Most incorrect classifications involved closely related or structurally overlapping loci in fragmented draft assemblies. Among 84 GBS-associated genomes with published LOS-class assignments, CjejuniTyper produced 83 concordant calls, corresponding to 98.8% concordance. In the Bangladesh case–control cohort, HIGH or MODERATE genomic profiles were identified in 34 of 38 GBS-associated isolates and 10 of 24 enteritis controls, corresponding to 89.5% sensitivity, 58.3% specificity, and 73.9% balanced accuracy. Across all 89 retained GBS-associated genomes, 81 (91.0%) received HIGH or MODERATE profiles. These profiles were enriched among GBS-associated isolates but were also present in some controls, indicating genomic mimicry-associated potential rather than patient-level clinical prediction. CjejuniTyper provides a reproducible workflow for integrated *C. jejuni* capsule and LOS typing with ganglioside-mimic, virulence, and antimicrobial-resistance annotations in a single output.

## 1 Introduction

*Campylobacter* is a leading cause of bacterial gastroenteritis worldwide, causing an estimated 96 million infections annually and accounting for 5–14% of diarrhoeal disease globally, with a disproportionate burden in low- and middle-income countries, particularly among children younger than 2 years (Cheng and Fischer, 2020; European Food Safety Authority (EFSA) and European Centre for Disease Prevention and Control (ECDC), 2025). *Campylobacter* infections typically present with fever, abdominal pain, and watery or bloody diarrhoea (Cheng and Fischer, 2020). However, while most people who contract *Campylobacter* infections will recover without treatment, some will experience long-term effects after their initial infection. Of these post-infection effects, perhaps the most dangerous include reactive arthritis, irritable bowel syndrome, and most seriously, Guillain-Barré syndrome. GBS is an acute autoimmune peripheral neuropathy and the leading cause of non-polio acute flaccid paralysis worldwide (Nachamkin et al., 1998). Based upon previous estimates, approximately one out of 1,000 individuals infected with *Campylobacter jejuni* (*C. jejuni*) develops GBS, and 20–50% of individuals diagnosed with GBS have evidence of a prior *C. jejuni* infection (Islam et al., 2009; Nayeem et al., 2025). The primary mechanism underlying this association is molecular mimicry. Certain *C. jejuni* strains express sialylated LOS structures on their outer membrane that structurally mimic human peripheral nerve gangliosides. As a result, IgG autoantibodies produced against LOS molecules cross-react with gangliosides found on human nerve cells and result in damage to those cells (Cheng et al., 2008; Parker et al., 2008). Because *C. jejuni* infections are predominantly sporadic and are commonly linked to poultry, cattle, or contaminated water, robust strain typing is essential for surveillance, outbreak investigation, and identification of GBS-associated strains.

The primary serodeterminant of *C. jejuni* is a CPS that covers the bacterium. The CPS biosynthetic genes are located within a large, highly variable segment of the chromosome, flanked by conserved ABC-transporter genes (*kpsC–kpsF*), and the variation in these genes results in at least 47 HS serotypes identified by the Penner agglutination system (Pike et al., 2013; Poly et al., 2011; Penner and Hennessy, 1980). These 47 serotypes represent approximately 35 different types of capsules and may be clustered into complexes of similar serotypes, many of which are structurally very close (i.e., >97% DNA similarity) (Pike et al., 2013; Poly et al., 2015). In addition to providing a protective barrier around the bacterial cell, CPS also plays critical roles in the pathogenicity of *C. jejuni*. For example, CPS mediates serum resistance to complement-mediated killing, facilitates the colonization of both avian and mammalian hosts, and is required for full invasiveness because CPS-deficient mutants display reduced ability to invade intestinal epithelial cells and display significantly lower levels of virulence compared to wild-type bacteria (Poly et al., 2011). Structural diversity is driven by gene gain and loss, phase variation at homopolymeric poly-G/C tracts, and the addition of unusual modifications such as O-methyl-phosphoramidate (MeOPN) and deoxy-heptoses, which can modulate host immune recognition and serotype within a complex (Pike et al., 2013; Poly et al., 2015).

Although Penner serotyping has proven useful in terms of defining many aspects of the epidemiology of *C. jejuni* infections, there are several limitations inherent to this method. First, the procedure is laborious. Second, the antisera used to define the various serotypes are expensive. Third, a proportion of the strains isolated during outbreaks and sporadic cases will fail to be typed using available antisera due to phase variation or limited availability of antisera to react with the epitopes present on certain strains (Pike et al., 2013; Rautelin and Hänninen, 1999). Other early molecular typing approaches developed in response to some of the limitations encountered with traditional serotyping techniques included restriction fragment length polymorphism (RFLP) analysis of LOS genes, pulsed-field gel electrophoresis (PFGE), and ribotyping. Although these techniques offered improved discriminatory power relative to traditional serotyping methods, they were not suitable for routine use in laboratories due to high costs and complexity (Owen et al., 1995; Nakari et al., 2005). Following the development of multiplex PCR assays designed to identify the genetic determinants responsible for encoding variable CPS loci, many of the traditional serotypic tests utilized in laboratories were eventually replaced (Poly et al., 2011; Poly et al., 2015). Initially, one of the earliest methods was capable of distinguishing between 17 different capsule types. Subsequent studies expanded the scope of this assay to include all 47 Penner serotypes (Poly et al., 2011; Poly et al., 2015). More recent reports indicate that additional CPS loci not previously detected using PCR-based methods have been discovered in strains recovered from individuals residing in South and Southeast Asia and thus indicate that continued updates to existing classification systems will likely be necessary (Khemnu et al., 2023). Although PCR-based methods offer advantages over traditional serotyping techniques, they remain laboratory- and culture-dependent, require separate assays for LOS class identification, and do not integrate virulence or antimicrobial-resistance profiling into a single workflow.

*C. jejuni* expresses LOS rather than a conventional long O-antigen lipopolysaccharide (LPS). The LOS biosynthesis gene cluster is one of the most genetically heterogeneous parts of the *C. jejuni* genome; it exhibits frequent recombination, horizontal gene exchange, and phase variation that give rise to at least 23 classes (A-W) that can be broadly categorized into four functional groups (Parker et al., 2008; Hameed et al., 2022). LOS group 1 classes (Classes A-B-C-M-R) contain sialic acid synthesis genes (*neuA-neuB-neuC*) and sialyltransferase (*Cst-II* or *Cst-III*) coding regions that enable sialic acid residues to be added to the LOS outer core structure, creating ganglioside-like structures that mimic human GM1, GD1a, GT1a, and similar gangliosides (Parker et al., 2008; Hameed et al., 2022). Cross-reactive antibodies produced in response to these LOS structures induce damage to peripheral nerve gangliosides, thereby inducing Guillain-Barré syndrome (GBS) (Islam et al., 2009; Cheng et al., 2008). Approximately 69% of GBS-associated isolates from Bangladesh carried LOS Classes A (32%) or B (37%) (Nayeem et al., 2025). LOS Class B predominates among strains associated with Miller Fisher syndrome. LOS Class R is a structural composite derived from Class A and contains sialylation genes; while it is considered sialylated, LOS Class R has less well-documented associations with GBS in clinical cohorts than Classes A, B, and C (Parker et al., 2008). Classes D–E and others lacking sialylation genes have been associated with uncomplicated enteritis (Hameed et al., 2022).

The fine specificity of ganglioside mimicry is further determined by a single amino acid polymorphism at position 51 of *Cst-II*: an asparagine residue (Asn51) renders the enzyme bifunctional, catalyzing both α2,3- and α2,8-sialyl linkages to produce disialylated GT1a and GD3 mimics associated with Miller Fisher syndrome, whereas a threonine residue (Thr51) yields a monofunctional enzyme that adds only a single α2,3-linked sialic acid, generating GM1 and GD1a mimics linked to the acute motor axonal neuropathy (AMAN) variant of GBS (Cheng et al., 2008; Parker et al., 2008). Together with the presence of accessory transferases encoded by *cgtA* and *cgtB*, this allelic variation at codon 51 supports putative inference of ganglioside-mimic biosynthetic potential from genome sequence. This inference represents genomic potential only and does not prove LOS expression or the chemical structure of the produced LOS.

Epidemiologic studies have found associations between certain capsular types and increased risk of Guillain-Barré syndrome (GBS). Certain types, including Penner serotypes HS19, HS41, HS23/36, HS4, HS2, and HS1/44, are identified more frequently in GBS cases than in enteritis cases caused by *C. jejuni* (Heikema et al., 2015; Wang et al., 2022). Among these, infection with an HS19 strain increases the risk of developing GBS (at least 6-fold) when compared to infection with strains expressing other Penner capsular types. In fact, approximately 67–75% of Japanese *C. jejuni* strains causing GBS express HS19 (Heikema et al., 2015). However, regional variability exists regarding which Penner capsular types are most commonly associated with GBS cases within specific geographic areas. For example, in Bangladesh the predominant Penner capsular type among GBS cases is HS23_36, whereas in China the predominant types are HS2, HS19, and HS41 (Islam et al., 2009; Parker et al., 2008; Wang et al., 2022). *C. jejuni* HS19 reference strain RM3420 carries both an HS19 capsule locus and a Class A LOS locus, illustrating the convergence of CPS and LOS risk signals in a single lineage (Parker et al., 2017). Therefore, simultaneously determining the serotype and LOS class of a *C. jejuni* isolate provides more comprehensive GBS-associated genomic information than determining either one separately.

Recent genomic studies analysed *C. jejuni* isolates already associated with GBS through clinical diagnosis or previously reported source information. Nayeem et al. (2025) analysed 38 isolates from patients with clinically diagnosed GBS and 24 enteritis controls from Bangladesh, while a subsequent international study expanded the collection to 90 GBS-associated genomes by including 52 additional isolates from public datasets (Hayat et al., 2026). In these studies, GBS status was not predicted from bacterial genome sequences. Instead, genome analysis was used to assign LOS classes and characterize other bacterial features. These datasets therefore provide an opportunity to evaluate the concordance of CjejuniTyper LOS assignments and the distribution of its GBS-associated genomic profiles among reported GBS-associated isolates and enteritis controls.

Whole-genome sequencing is now routinely applied in clinical and public health microbiology laboratories, providing genome assemblies for tens of thousands of *C. jejuni* isolates. Computational tools have emerged for multilocus sequence typing (MLST) and antimicrobial-resistance prediction, but, to our knowledge, no publicly available pipeline simultaneously performs capsule typing, LOS class assignment, *Cst-II* allele calling, virulence-gene profiling, and interpretation of GBS-associated genomic markers from genome assemblies. This unmet need motivated the development of CjejuniTyper as an integrated, reproducible, and openly available computational workflow for genome-based characterization of *C. jejuni*. Multiplex PCR methods do not type LOS classes or assess *Cst-II* alleles; MLST identifies clonal lineages but does not directly predict surface antigen structure or neuropathogenic potential, and binary gene typing panels provide discriminatory power for outbreak investigations without linking to GBS risk (Huang et al., 2013). One earlier study applied *in silico* PCR to CPS locus sequences for capsule typing, but this approach addressed capsule typing only, did not extend to LOS classification, and was not implemented as an integrated genome analysis workflow (Khemnu et al., 2023). Systematic reviews document that eight capsule types account for more than 50% of global *C. jejuni* infections, underlining the importance of scalable, reproducible surveillance tools for vaccine development efforts (Pike et al., 2013). The continual discovery of novel CPS loci further highlights the advantages of a BLAST-based genomic approach over PCR schemes with fixed primer sets (Khemnu et al., 2023).

In this study, we describe CjejuniTyper, a computerized bioinformatics pipeline designed to utilize well-established genomic markers and curated reference sequences to predict CPS complexes and LOS classes from *C. jejuni* whole-genome assemblies. Additionally, the pipeline identifies *Cst-II* alleles and additional sialylation-related genes, predicts possible ganglioside-mimic biosynthesis capabilities, detects virulence-related genes, and incorporates antimicrobial-resistance results generated by AMRFinderPlus. Penner CPS and LOS assignments were evaluated against curated datasets comprising 96 *C. jejuni* assemblies with known Penner types and 617 assemblies with known LOS classes, respectively. The integrated GBS-associated genomic classification was additionally evaluated using 113 retained genomes comprising 89 isolates previously reported from GBS patients and 24 enteritis controls. Ganglioside-mimic and GBS-associated classifications are provided for informational and surveillance purposes, while all typing, virulence, and antimicrobial-resistance results are reported together in a single consolidated output. The proposed workflow complements existing serological and molecular typing strategies and enables the derivation of several important clinically and epidemiologically relevant features from a single *C. jejuni* genome assembly. The workflow is freely available via GitHub^1^ to facilitate reproducible genomic analyses and public health surveillance.

## 2 Materials and methods

### 2.1 Pipeline overview

CjejuniTyper is a modular bioinformatic pipeline implemented as a Bash shell script that contains embedded Python 3 modules. This tool accepts whole-genome assemblies in FASTA format (.fa, .fasta, or .fna) and generates a single tab-delimited output file (final_los_hs_gbs.tsv) containing integrated typing, virulence, resistance, and GBS-associated genomic profiling data for each isolate (Figure 1). The pipeline has seven stages that are executed sequentially: assembly quality control, Mash species confirmation, MLST typing, Penner (HS) capsule typing, LOS class typing, virulence-factor detection, and antimicrobial-resistance profiling. Its primary analytical functions consist of the following:

- Species confirmation. Mash v2.3 is used to screen each input assembly against a reference sketch containing *C. jejuni* and *C. coli* genomes before *C. jejuni*-specific typing. The reference with the highest shared-hash ratio is selected when the shared-hash ratio exceeds 0.5. Assemblies classified as *C. coli* or species-uncertain remain visible in the output but are not subjected to downstream *C. jejuni*-specific typing (Ondov et al., 2016).
- Penner CPS typing. BLASTN searches against a curated database of serotype-specific marker genes are used to assign a capsule type or complex. Full CPS locus alignment against reference sequences is performed for confirmed *C. jejuni* assemblies and provides fallback and supporting evidence for Penner type assignment. Selected complexes are further resolved using poly-G/C tract analysis for HS23_36c, amino-acid BLASTX analysis for HS8_17c, and *in silico* PCR confirmation for selected types, including HS29, HS55, and HS58.
- LOS class and *Cst-II* allele analysis. LOS classes are assigned using a best-hit BLAST scoring system with defined nucleotide identity and coverage thresholds. Codon 51 of *Cst-II* is examined to distinguish the bifunctional Asn51 and monofunctional Thr51 variants. Sialylation-associated and accessory transferase genes, including *cgtA, cgtB, neuB, and wlaN*, are detected to infer putative ganglioside-mimic biosynthetic potential.
- Virulence and antimicrobial-resistance profiling. BLAST searches against a curated virulence-factor database identify genes associated with capsule and LOS biosynthesis, cytolethal distending toxin, capsule modification, and the Type IV secretion system. AMRFinderPlus is used to detect acquired antimicrobial-resistance genes and resistance-associated point mutations.
- Integrated interpretation. A rule-based interpretation engine combines LOS sialylation status, Penner GBS-associated serotypes, *Cst-II* allele status, and putative ganglioside-mimic genotype to produce a three-tier GBS-associated genomic classification. These classifications are intended for research and surveillance and not as patient-level clinical predictions.

**Figure 1:**
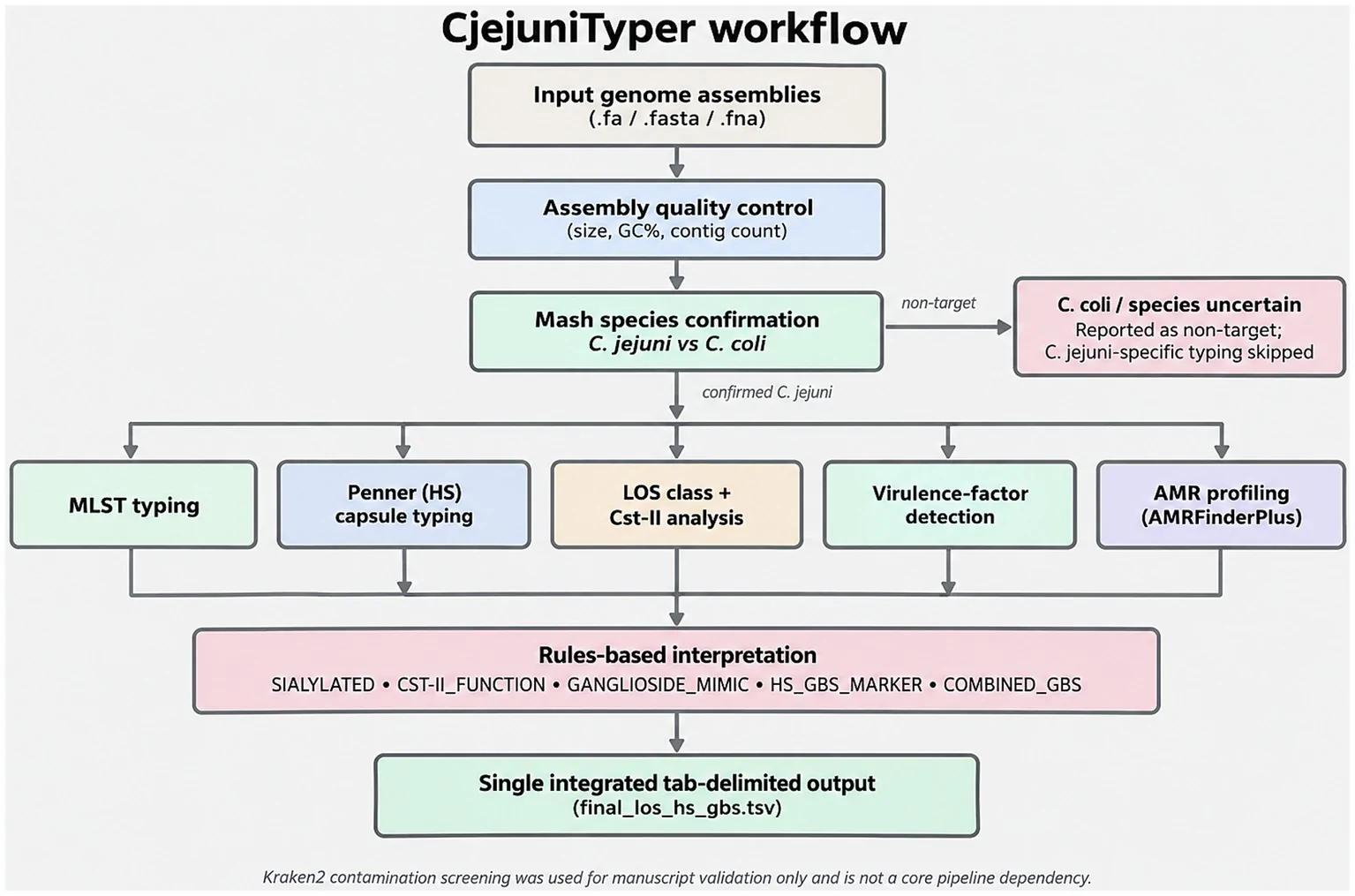
CjejuniTyper workflow. Input genome assemblies first undergo basic assembly quality control followed by Mash species confirmation. Assemblies confirmed as *C. jejuni* proceed to MLST, Penner (HS) capsule typing, LOS/*Cst-II* analysis, virulence-factor detection, and AMR profiling; *C. coli* or species-uncertain assemblies are retained in the reports but *C. jejuni*-specific typing is skipped. The typing and marker outputs feed a rule-based interpretation engine that derives sialylation status, *Cst-II* function, ganglioside-mimic, capsule and LOS GBS markers, and a combined GBS-associated genomic score. All results are merged into a single output file. Kraken2 contamination screening was used for manuscript validation only and is not a core pipeline dependency.

The final interpretation engine incorporates all of the module outputs into a single summary table and computes derived columns including SIALYLATED, *CST-II*_FUNCTION, GANGLIOSIDE_MIMIC, GBS_MIMIC_RISK, HS_GBS_MARKER, and COMBINED_GBS. Individual modules can be skipped using dedicated command-line options. Additionally, the pipeline can be executed in parallel across many samples utilizing a configurable “jobs” parameter. The CjejuniTyper workflow is illustrated in Figure 1. CjejuniTyper is freely available at https://github.com/Gagi1993/CjejuniTyper and permanently archived on Zenodo (https://zenodo.org/records/22871020 DOI: https://doi.org/10.5281/zenodo.22871020). The pipeline is written as a Bash shell script containing embedded Python 3 modules, and it is designed to run on Linux platforms. Installation can be accomplished using the bundled Conda/Mamba environment. The software is distributed under the MIT License.

Required dependencies include NCBI BLAST+ version 2.13 or later, NCBI AMRFinderPlus, Exonerate with the ipcress module, SeqKit, and Mash v2.3. All of these packages can be installed through the bundled Conda/Mamba environment named cjejuni_typer (Camacho et al., 2009; Shen et al., 2024; Slater and Birney, 2005; Feldgarden et al., 2021; Wood et al., 2019). An example usage would be as follows:

bash CjejuniTyper.sh -i assemblies/ -o results/ -t 32 -j 4

Before BLAST analysis, input FASTA files were normalized to remove carriage returns and blank lines. When SeqKit was available, it was also used to re-wrap and validate FASTA records to prevent parsing errors caused by non-standard sequence wrapping.

### 2.2 Genome dataset acquisition, assembly quality control, and species confirmation

The whole-genome assemblies of *C. jejuni* were retrieved from the NCBI Assembly database via the NCBI Datasets command-line tool (O’Leary et al., 2024). All assemblies were downloaded in FASTA format, and their respective metadata were also retained for later traceability. The accession identifier and the name of each strain were saved in tables as links to both the reference label for validation of LOS classes and Penner types.

CjejuniTyper performs a number of preliminary assessments regarding the quality and species identity of an input genome. Basic assembly quality control (QC) computes total assembly size, contig count, and GC content directly from the FASTA sequence. A “WARN” flag is placed on an assembly when either (I) it has an assembled genome length outside of the normal range for *C. jejuni* (1.3–2.0 Mb), (II) it has a GC percentage outside the normal range for *C. jejuni* (27.5–33.5%), or (III) it has more than 500 contigs. The QC results are written to qc_report.tsv, and warnings do not automatically exclude an assembly. Species confirmation is then performed with Mash (Ondov et al., 2016) v2.3 using mash screen against a sketch containing *C. jejuni* NCTC 11168 and *C. coli* FDAARGOS_735 references. A reference is considered supported when the shared-hash ratio is greater than 0.5, and the supported reference with the highest ratio determines the species call. Assemblies classified as *C. jejuni* proceed to downstream typing; *C. coli* and species-uncertain assemblies are reported as non-target and *C. jejuni*-specific analyses are skipped (Ondov et al., 2016).

For validation of the manuscript datasets, an additional broad contamination screen was performed separately with Kraken2 using the MiniKraken2 v2 8GB 201904 database (Wood et al., 2019). Kraken2 was not added as a required dependency of the core CjejuniTyper workflow. For assemblies in which the Kraken2 top-species call was discordant with Mash, the raw Kraken2 output was reviewed at the base-pair level by summing the lengths of contigs assigned to the principal non-*Campylobacter* taxon and to *C. jejuni*. This allowed substantial mixed-species contamination to be distinguished from small numbers of short contaminant contigs.

### 2.3 Construction of reference databases

A number of different nucleotide BLAST databases have been created specifically for LOS-class typing, Penner HS capsule typing, virulence-factor detection, and detection of auxiliary markers. The databases were then formatted for use with BLAST by running them through the makeblastdb program that comes with NCBI BLAST+.

The LOS reference database has been generated from the 19 LOS-class reference sequences (Classes A–S) which had previously been compiled and published by Hameed et al. (2022) who defined the LOS classes through the use of a Megablast alignment of 703 *C. jejuni* genome sequences against reference loci spanning the entire biosynthetic region from *waaM* to *waaV*. The GenBank accession numbers for each LOS-class reference are presented in Table 1. All of the 19 class reference sequences were combined together into one large BLAST database. An additional class-specific marker database for LOS Class B was generated from non-overlapping 1-kb sliding windows across the *C. jejuni* 81–176 LOS B locus (positions 1–17,000) and was used as additional evidence for distinguishing between Classes A and B when their scores were very similar. In addition, a unique database was prepared using only the *orf11* gene sequence to aid in A/R discrimination.

**Table 1:** GenBank reference sequences used for LOS class assignment in CjejuniTyper, from Hameed et al. (2022).

LOS class	Reference accession
A	AF215659
B	Cj81-176
C	NCTC11168
D	AF400669
E	EU404105
F	EU404108
G	AAR98510
H	EU404106
I	EU404107
J	EU404104
K	EF143353
L	EU404111
M	EF140720
N	AY816330
O	EF143352
P	AY943308
Q	EU404112
R	AY962325
S	EU404110

The Penner HS capsule typing database was developed from two sub-databases: a marker-gene database and a full-locus reference database. These reference sequences were obtained from capsule locus accessions reported by Poly et al. (2011) and Liang et al. (2016). The database contains 31 primary reportable reference entries, including both individual HS types and established serotype complexes (Table 2). The underlying CPS database contains 35 non-duplicate reference labels, corresponding to the established collapsed capsule-type representation of the 47 recognized Penner HS serotypes. CjejuniTyper reports these as 31 conservative HS categories and complexes because several Penner serotypes share highly similar CPS loci and cannot be reliably separated from genome assemblies alone. However, where sufficient genomic information exists for subtype determination within a complex, the determined subtype will appear in brackets, i.e., HS8_17c(HS8), HS45c(HS45*), HS23_36c(HS36), or HS4c(HS16/64*). The * indicates that this represents a predicted genomic subtype and not a proven serological type. Thus, the main CjejuniTyper HS designation remains the complex name; however, any bracketed subtypes present can provide additional resolution within certain complexes.

**Table 2:** GenBank accession numbers of CPS (Penner HS) reference sequences used in the CjejuniTyper capsule typing database, derived from Poly et al. (2011), Liang et al. (2016).

Penner HS type/complex	Represents HS types if complex	GenBank accession(s)
HS1c	HS1, HS44	BX545859/LISM00000000
HS2	—	AL111168/NC_002163
HS3	—	HQ343268
HS4c	HS4, HS13, HS43, HS50, HS62, HS65 may be bracketed as HS4c(HS4*) or related HS4-complex subtype when supported	HQ343269/AASY01000000
HS4c/B	HS4c internal bracket — HS16, HS64; reported as HS4c(HS16/64*) when Mu_HS4B PCR is positive	HQ343269/AASY01000000
HS5_31c	HS5, HS31	KT868847
HS6_7c	HS6, HS7	NC_009839
HS8_17c	HS8, HS17	HQ343270/HQ343273
HS9	—	KT868844
HS10	—	HQ343271
HS12	—	ABGQ01000005
HS15c	HS15, HS58	HQ343272
HS19	—	BX545860
HS23_36c	HS23, HS36	BX545858/AY332624 / AY332625
HS41	—	BX545857/ATBJ00000000
HS42	—	HQ343274
HS53	—	ATBN00000000
HS11	—	KT868845
HS18	—	KT932997
HS21	—	KT868849
HS22	—	KT893439
HS27	—	KT893437
HS29	—	KT868846
HS37	—	KT893431
HS38	—	KT893430
HS40	—	KT893434
HS57	—	KT893428
HS45c	HS45, HS32, HS60	KT893432/KT893435/KT893426
HS33_35c	HS33, HS35	KT893436
HS52	—	KT893429
HS55	—	KT893433
HS63	—	KT893438

Complex entries are reported by CjejuniTyper at the complex level; listed member serotypes indicate the serotypes represented by that complex call, not separate independent reference entries.

The full-locus database contained whole CPS locus reference sequences that were downloaded from NCBI GenBank using the indicated accessions; sequences for serotypes not covered by these resources were obtained from PubMLST (Jolley and Maiden, 2010). Amino-acid BLASTX, poly-G/C tract analysis, and *in silico* PCR confirmation using ipcress were used for subtype prediction within selected complexes, based on the updated multiplex typing scheme of Poly et al. (2015).

In Table 2, complex designations are representative of the main CjejuniTyper HS call. Bracketed labels representing subtype predictions within a complex (when produced) are labeled with an asterisk (*). Bracketed labels are intended to represent the best efforts of genomic predictions within a given complex and should not be considered as validated Penner serotypes. For example, an isolate that is designated as belonging to the HS4 complex is referred to primarily as HS4c; if additional markers allow for resolution of the subtype, the output may contain a bracketed prediction such as HS4c(HS16/64*). Within the HS4 complex, two genomic reference subgroups were used: HS4A, comprising HS4, HS13, HS43, HS50, HS62, and HS65, and HS4B, comprising HS16 and HS64. Both subgroups are reported primarily as HS4c, with HS4B optionally bracketed as HS4c(HS16/64*); these subgroup designations do not represent separate Penner serotypes.

The virulence-factor reference sequences for virulence-factor detection were downloaded from VFDB and processed to retain only *Campylobacter* relevant markers (Chen et al., 2016). Databases were created for general virulence factors; *Cst-II* alleles; Type IV Secretion System Subunits; and *orf11*.

### 2.4 MLST typing

MLST of *C. jejuni* isolates was performed using the seven-gene PubMLST scheme comprising *aspA*, *glnA, gltA, glyA, pgm, tkt,* and *uncA* (Jolley and Maiden, 2010; Dingle et al., 2001). For each locus, genome assemblies were searched using BLASTN against a local database of PubMLST allele sequences. Candidate alleles were required to have ≥99% nucleotide identity and ≥90% allele coverage, and the highest-scoring qualifying allele was retained for each locus. Allele numbers were extracted from the reference sequence identifiers. When all seven loci were resolved, the resulting allelic profile was compared with a local copy of the PubMLST profile table to assign sequence type (ST) and clonal complex (CC). Complete seven-allele profiles that were not present in the profile table were reported as novel, whereas isolates with one or more unresolved alleles were reported as unresolved.

### 2.5 LOS class typing

Each assembly was compared by BLASTN against the combined LOS database using an E-value threshold of ≤1 × 10^−10^. Only BLAST hits with ≥78% nucleotide identity were retained. The raw BLAST hits were grouped by allele to account for assemblies containing multiple contigs. The hits belonging to each allele were combined, and the best allele for each LOS class was selected based on the total aligned length. Assignment of a LOS class follows a best-hit approach based upon the product of percentage identity and fractional coverage, without bit-score weighting to avoid reference-length bias. Seven decision tiers were defined in decreasing order of stringency: STRICT (≥99.5% identity; ≥96% coverage), STRICT_2 (≥99.0%/95%), STRICT_3 (≥97.0%/93%), STRICT_4 (≥95.0%/93%), MID (≥94.0%/80%), MID_2 (≥92.0%/80%), and LOOSE (≥90.0%/60%). The thresholds were selected empirically during pipeline development by examining BLAST identity and coverage patterns for assemblies with previously reported LOS classes. Because several LOS loci are highly similar, identity and coverage were considered jointly and the tier boundaries were chosen to retain the expected class assignment while discriminating closely related reference loci. The tiers are evaluated hierarchically, with the highest-stringency qualifying evidence preferred; lower tiers act as fallback criteria when a locus is only partially represented, as can occur in fragmented draft assemblies. Thus, a LOOSE-tier hit is not considered equivalent to a near-complete STRICT-tier hit. To limit classification from weak partial matches, assemblies falling below the LOOSE threshold are reported as untypeable, and hits with 60–68% coverage are also rejected when nucleotide identity is <97%.

The LOS class with the highest score (Hameed et al., 2022) is assigned to each assembly. For cases where the Class A and Class B scores differ by no more than 5%, a close-call rule based on the difference in total aligned bases is applied. In this manner, if Δ(B_ALN - A_ALN) is greater than 5,000 bp, then Class B is assigned; otherwise, Class A is assigned. Similar close call rules based upon normalization for bitscore are applied to H/P and S/J pairs. To distinguish between Class A and Class R calls at non-STRICT tiers, a final *orf11* BLAST step is performed: if *orf11* is found with ≥ 90% identity and ≥ 80% coverage, then Class A is assigned; if not present, Class R is assigned. Finally, an additional step for identifying *neuA1* by the virulence-factor module will be performed after calling: If *neuA1* is detected, then the LOS class will be overridden and the type called as Class C.

### 2.6 Penner (HS) capsule type prediction

*In silico* Penner serotyping was performed as a four-step system covering 31 CPS reference types and serotype complexes. In classical Penner serotyping, serologic assays rely on antibodies to detect specific HS antigens. Here, the antibody based assay has been replaced by genomic detection of HS-associated sequence markers and entire CPS loci (Penner and Hennessy, 1980).

Step 1—Marker-gene BLAST: Assemblies were screened against the HS marker-gene database using BLASTN. Marker hits were evaluated using three hierarchical decision tiers: Tier A (≥99.5% identity and ≥80% coverage), Tier B (≥95%/75%), and Tier C (≥90%/60%). These thresholds were established empirically during method development by comparing BLAST identity and coverage patterns with the previously reported Penner types of the assemblies and selecting boundaries that discriminated the expected marker set from closely related alternatives. The highest-stringency qualifying tier was considered first, and lower tiers were used only when higher-tier evidence was unavailable. Lower coverage thresholds permit marker recovery from fragmented draft assemblies in which an otherwise highly similar marker may be interrupted by a contig boundary. The serotype with the greatest number of marker-gene hits at the highest available tier was assigned; where multiple candidates had equal numbers of hits, ties were resolved using mean nucleotide identity. Only Tier A hits were accepted for HS19 because its primary marker gene cross-reacts with neighboring serotypes at lower identity thresholds. Marker-gene assignments take precedence during the Step 1/Step 2 merge, except for curated cross-reactive conflict rules, after which applicable subtype-bracketing and *in silico* PCR rules are evaluated.

Step 2—Full-locus BLAST fallback: Full-locus BLAST was performed for all confirmed *C. jejuni* assemblies against the full CPS locus database; the full-locus result served primarily as a fallback for samples unresolved by Step 1 and also provided supporting evidence for conflict resolution and subtype interpretation. Hits with ≥3,000 base pairs of total aligned length were evaluated using percent identity x fractional coverage x bitscore. The top scoring serotype complex was assigned. When the two highest-scoring serotype complexes were very similar, with the difference in percent identity within 0.5% and the difference in coverage within 10%, and they were not a recognized cross-reactive pair, both were reported together as a combined call (e.g., HS9_HS10) to indicate ambiguity for manual review. Additionally, the winner information generated during full-locus BLAST evaluation was used to generate bracketed genomic subtypes when supported for the HS45-associated group (e.g., HS45c(HS45*), HS45c(HS32*), or HS45c(HS60*)).

Step 3—Subtype bracketing: Two complexes needed additional resolution. For HS8_17c, this was achieved by translating BLASTX against amino acid databases for HS8 and HS17; in cases where the amino acid databases identified ≥3 matching positions with a greater score than the other database, then the type was denoted in brackets as HS8_17c(HS8) or HS8_17c(HS17). For HS23_36c, this was accomplished by evaluating the longest homopolymeric G or C tract within the poly-G/C region of the *wlaN/cj1295c* transferase gene; if the tract length is divisible by three, then it indicates a frameshifted gene (→ HS23); otherwise, it is indicative of an in-frame functional gene (→ HS36). An asterisk (*) represents a predicted subtype that has not been verified by serology.

Step 4—*In silico* PCR was performed using ipcress from the Exonerate package with primers derived from previously described molecular subtyping schemes (Poly et al., 2011; Poly et al., 2015); override rules included HS29 (product size 150–250 bp), HS55 (300–400 bp), and HS15c(HS58) (HS58_target 70–120 bp is co-present with HS15_target 300–400 bp). Bracket rule designations for complexes were HS4c(HS16/64) (HS4B product 625–680 bp), HS5_31c(HS31) (HS5_31 830–890 bp plus HS15 300–400 bp), and HS5_31c(HS5) (HS5_31 product alone).

Serotype complexes were reported conservatively. In cases where genetic data failed to differentiate among the various components of a given serotype complex, the overall complex designation remained without being bracketed. Bracketed subtype designations are representations of putative genetic subtypes and should not be considered equivalent to empirical evidence obtained via antiserum testing.

### 2.7 *Cst-II* allele typing and ganglioside-mimic prediction

The *Cst-II* gene was detected by BLASTN against a standalone *Cst-II* reference database (≥90% identity, ≥60% coverage, E-value ≤1 × 10^−10^, max one HSP). For positive hits, the codon at position 51 of the *Cst-II* protein was determined directly from the query assembly by extracting the aligned query region and translating the relevant triplet, accounting for strand orientation. Codons AAT or AAC were assigned as Asn51 (bifunctional enzyme); ACT, ACC, ACA, or ACG as Thr51 (monofunctional enzyme). Absent *Cst-II* was recorded as ABSENT.

Ganglioside-mimic type was predicted using a rule-based engine integrating: (i) presence of any sialylation gene (*Cst-II, Cst-III, neuA1, neuB, neuB1, neuC, neuC1*); (ii) *Cst-II* functional status; and (iii) accessory transferases *cgtA, cgtB*, and *wlaN*. The prediction logic was as follows: no sialylation genes → NON-SIALYLATED (LOW risk); sialylation genes present but no sialyltransferase → NO_TRANSFERASE (LOW_UNCERTAIN); bifunctional (Asn51) + *cgtA* + *cgtB* → GT1a (HIGH); bifunctional otherwise → GD3 (MODERATE); monofunctional (Thr51) + *cgtA* + *cgtB* → GM1/GD1a (HIGH); monofunctional otherwise → GM3 (MODERATE); *Cst-II* present but codon 51 unreadable → GM1/GD1a|GT1a unresolved if *cgtA* + *cgtB* present (HIGH, uncertain), otherwise unresolved sialylated LOS (MODERATE); *Cst-III* + *wlaN* → GM2/GM1 (MODERATE); *Cst-III* without *wlaN* → GM3 (MODERATE) (Figure 2). Because GM1 and GD1a are produced by the same gene set, they cannot be resolved by gene content and are reported jointly; the predicted mimics represent genomic potential and are informational only, not clinically validated predictions.

**Figure 2:**
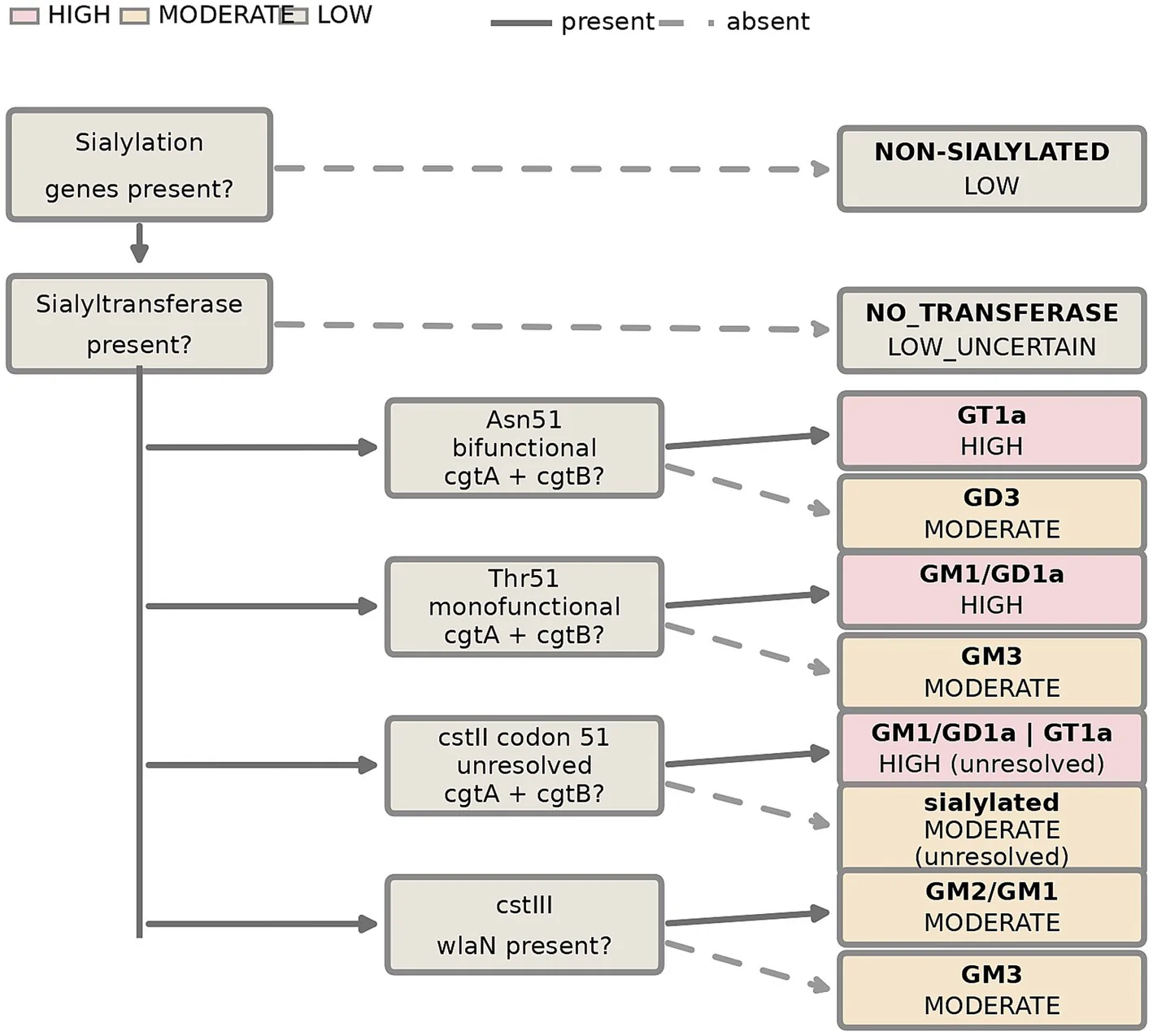
Decision logic for ganglioside-mimic prediction in CjejuniTyper. Branching is driven by two genomic switches: the *Cst-II* codon-51 allele (Asn51, bifunctional, α2,3/α2,8; or Thr51, monofunctional, α2,3 only) or the presence of *Cst-III*, and the chain-extension genes *cgtA* + *cgtB* (or *wlaN* on the *Cst-III* path). Solid branches indicate the feature is present; dashed branches indicate absence. Box color denotes the genomic mimic-risk tier. GM1 and GD1a are reported jointly because they derive from the same gene set and cannot be resolved by gene content; where codon 51 cannot be read, the call is left unresolved and flagged. Predicted mimics represent genomic potential inferred from the LOS biosynthesis genotype and are informational only; they are not clinically validated predictions of GBS in any individual.

### 2.8 Virulence-factor detection

Virulence-factor gene sequences were identified using BLASTN against a filtered database derived from VFDB (≥ 90% identity and ≥ 60% coverage). Reported gene types included sialylation genes (*Cst-II, Cst-III, neuA1, neuB, neuB1, neuC*, and *neuC1*); additional transferases (*cgtA, cgtB,* and *wlaN*) involved in LOS synthesis; LOS core biosynthetic genes (*gmhA, gmhA2, gmhB, hddA, hddC, hldD, hldE, rfaD, rfaE1, waaC,* and *waaF*); capsule biosynthesis/transport genes (*kpsC, kpsD, kpsE, kpsF, kpsM, kpsS,* and *kpsT*); cytolethal distending toxins (*cdtA, cdtB,* and *cdtC*); MeOPN modification genes (MEOP); and type-IV secretion system components (*virB2*–*virB11* and *virD4*). T4SS status was defined as COMPLETE if all 11 subunit sequences were present; PARTIAL if some but not all subunits were detected; and ABSENT if none were found. Similarly, CDT status was classified as COMPLETE when *cdtA, cdtB,* and *cdtC* were all detected; PARTIAL when only a subset of these three genes was detected; and ABSENT when none of the three genes was detected. Inferred serum resistance was determined by whether all seven CPS biosynthetic genes and all 11 LOS core biosynthetic genes were detected (YES), partially detected (PARTIAL), or undetected (NO). Due to the potential for certain plasmid-associated markers to be missing from incomplete draft genomes, caution should be exercised when interpreting the absence of T4SS components.

### 2.9 Antimicrobial-resistance profiling

Antimicrobial-resistance profiling was performed using NCBI AMRFinder Plus with the --organism *Campylobacter* --plus flags. AMRFinder Plus detects acquired resistance genes (*tet(O)*, *blaOXA* variants, *ant(6)-Ia, aph(3′)-IIIa*), the intrinsic multidrug efflux pump *cmeABC*, and clinically relevant point mutations: *gyrA* T86I, D90N, and P104S for fluoroquinolone resistance; *23S rRNA* A2075G and A2074C for macrolide resistance. STRESS category genes (e.g., arsenic resistance) were excluded from the final output. Each unique AMR determinant detected across all samples was reported as a separate column in the output table with percent identity and percent coverage values.

### 2.10 Integrated GBS risk scoring

A combined GBS risk score (COMBINED_GBS) was computed by integrating three genomic signals, with the ganglioside-mimic prediction as the primary determinant. The ganglioside-mimic risk (GBS_MIMIC_RISK) was derived from the predicted mimic type as described in Section 2.7. The LOS sialylation signal was taken from the SIALYLATED column: LOS classes A, B, C, M, and R were classified as sialylated, based on their encoding of the sialic acid biosynthesis cassette, and all other classes as non-sialylated (Parker et al., 2008; Hameed et al., 2022). The capsule signal (HS_GBS_MARKER) flagged Penner types HS1, HS2, HS4, HS19, HS23_36, and HS41 as GBS-marker serotypes based on established epidemiological evidence; each Penner call was first reduced to a canonical reference name so that serotype complexes and bracketed sub-calls were matched correctly (Heikema et al., 2015; Wang et al., 2022).

The combined score was then assigned hierarchically. A genome was scored HIGH when it carried a confirmed high-risk mimic genotype (GT1a or GM1/GD1a) together with a sialylated LOS class; where the mimic genotype was unresolved (Cst-II codon 51 unreadable) or of moderate risk (e.g., GD3 or GM3), a HIGH score additionally required both a sialylated LOS class and a GBS-marker serotype. Genomes carrying a mimic-type signal that did not meet these criteria—for example, a high-risk genotype without a sialylated LOS class, or a moderate genotype without a corroborating GBS-marker serotype—were scored MODERATE, as were genomes with a GBS-marker serotype and sialylated LOS but no ganglioside-mimic signal; all remaining genomes were scored LOW. A sialylated LOS class was therefore required for any HIGH assignment. These categories represent genomic risk indicators and are not intended as clinical diagnostic predictions.

### 2.11 Validation dataset and statistical evaluation

Across the LOS, Penner, and GBS-associated validation collections, 812 unique assemblies were screened for species identity with Mash and for broad contamination with Kraken2. Species and contamination QC decisions were recorded in Supplementary Table S1. Assemblies identified as non-target species or showing substantial non-*Campylobacter* contamination were excluded from the relevant performance calculations, while all screened accessions were retained in the Supplementary material for transparency.

Pipeline performance was evaluated on two curated validation datasets. For LOS class typing, 617 *C. jejuni* genome assemblies with known LOS class annotations were used. Reference LOS class labels were obtained from Hameed et al., who assigned LOS classes to 703 *C. jejuni* genome sequences in GenBank using Megablast alignment against LOS class reference sequences; assemblies with known class labels available in NCBI GenBank at the time of database construction were included (Hameed et al., 2022). The validation set comprised 125 complete genome sequences and 492 draft assemblies spanning all 19 LOS classes. Accession numbers are provided in Supplementary Table S1.

For Penner capsule typing, the initial collection contained 97 genome assemblies with known Penner type annotations. One assembly (108893_YZU2595) was identified as *C. coli* by both Mash and Kraken2 and was excluded from the *C. jejuni* typing evaluation, leaving 96 *C. jejuni* assemblies. Reference serotype labels were obtained from published molecular typing studies and from curated metadata in PubMLST (Poly et al., 2011; Poly et al., 2015; Nakari et al., 2005; Khemnu et al., 2023; Liang et al., 2016). Isolates with PubMLST numeric identifiers were retrieved directly from PubMLST (Jolley and Maiden, 2010); isolates with NCBI GCF/GCA accessions were retrieved from NCBI GenBank. CjejuniTyper reports results at the complex level rather than individual serotype level because several Penner serotypes share highly similar CPS loci and cannot be reliably distinguished by genome-based methods alone. For example, HS4c encompasses HS4, HS13, HS16, HS43, HS50, HS62, HS64, and HS65, with HS16 and HS64 reported only as an internal bracketed prediction when supported; HS5_31c covers HS5 and HS31; HS1c covers HS1 and HS44; and HS23_36c covers HS23 and HS36. Where NCBI or PubMLST metadata recorded individual serotype members rather than the complex designation used by CjejuniTyper, reference labels were manually harmonized to the complex level prior to evaluation (e.g., HS23 → HS23_36c and HS5 → HS5_31c). The retained validation set covered 14 of the 31 implemented CPS reference entries, collectively representing 27 individual Penner serotypes. The remaining 17 implemented types were not represented by assemblies with verified reference labels, and their accuracy therefore remains to be formally established. Accession numbers, species-QC results, and reference labels are provided in Supplementary Table S1.

The GBS-associated genomic classification was initially evaluated using 114 genome assemblies comprising 90 isolates previously reported from GBS patients and 24 enteritis controls (Nayeem et al., 2025; Hayat et al., 2026). One external GBS assembly (GCA_021890035.1) was excluded from performance calculations after Kraken2 base-pair review showed 219,589 bp (11.723% of the assembly) assigned to *Enterococcus faecium*, leaving 113 retained genomes comprising 89 GBS-associated isolates and 24 controls. Two other Kraken2 top-species discordances were retained because the non-*Campylobacter* fractions were minor: GCA_021890115.1 contained 14,071 bp (0.859%) assigned to *Vibrio furnissii* and GCF_002178815 contained 25,878 bp (1.474%) assigned to *Acinetobacter* spp.; Mash classified both as *C. jejuni*. Clinical-group labels were obtained from the source studies and were not inferred from genome sequences. Published LOS classes were compared with CjejuniTyper predictions using exact class agreement, excluding isolates with undetermined published LOS classes and the QC-excluded assembly. For evaluation of the integrated classification, HIGH and MODERATE results were considered positive genomic profiles, whereas LOW results were considered negative. The Bangladesh case–control cohort was used to calculate sensitivity, specificity, accuracy, balanced accuracy, precision, negative predictive value, F1-score, Matthews correlation coefficient, odds ratio, and Fisher’s exact test. The retained external GBS-associated genomes were analysed separately as a detection-rate analysis because no corresponding external control group was available. Accession numbers, QC decisions, and reference labels are provided in Supplementary Table S1.

As a *post-hoc* investigation of discordant control classifications, protein-coding sequences were predicted with Prodigal (Hyatt et al., 2010) and screened by BLASTP against curated *Cst-II, CgtA, CgtB, NeuB,* and *NeuC* reference proteins to assess whether the mimicry-associated pathway appeared complete.

Performance was quantified by overall accuracy, per-class precision, recall, and F1-score, macro-averaged and weighted F1-scores, Cohen’s kappa coefficient (κ), Matthews correlation coefficient (MCC), and 95% confidence intervals for accuracy estimated by the Wilson score method (Wilson, 1927). Confusion matrices were generated to characterize misclassification patterns. All metrics were computed using a custom Python script.

## 3 Results

### 3.1 Validation dataset quality control and species confirmation

Across the three validation collections, 812 unique assemblies were screened. Mash classified 811 as *C. jejuni* and one (108893_YZU2595) as *C. coli*; the *C. coli* assembly was excluded from the Penner evaluation. Kraken2 top-species screening identified three additional assemblies with non-*Campylobacter* top calls. Base-pair-level review showed substantial contamination in GCA_021890035.1, with 219,589 of 1,873,142 bp (11.723%) assigned to *Enterococcus faecium*; this assembly was excluded from the GBS-associated evaluation. The other two assemblies contained only minor non-*Campylobacter* fractions—0.859% *Vibrio furnissii* in GCA_021890115.1 and 1.474% *Acinetobacter* spp. in GCF_002178815—and were retained. No additional species-uncertain or mixed *C. jejuni*/*C. coli* assemblies were identified by Mash.

### 3.2 LOS class validation

The LOS class validation dataset comprised 617 *C. jejuni* genome assemblies spanning all 19 LOS classes (A–S), sourced from Hameed et al. (2022) and retrieved from NCBI GenBank. The dataset included 125 complete genome sequences and 492 draft assemblies. Classes B (*n* = 170), C (*n* = 113), and A (*n* = 79) were the most represented, reflecting their known prevalence in sequenced *C. jejuni* collections; rare classes D, N, and Q were each represented by a single assembly. The class distribution is shown in Table 3.

**Table 3:** Distribution of LOS classes in the validation dataset (*n* = 617).

LOS class	*n*
A	79
B	170
C	113
D	1
E	16
F	8
G	7
H	68
I	3
J	3
K	16
L	6
M	7
N	1
O	63
P	43
Q	1
R	6
S	6

CjejuniTyper correctly assigned LOS class for 595 of 617 genome assemblies, yielding an overall accuracy of 96.4% (Table 4). Agreement with reference labels was high, with Cohen’s κ = 0.958 and MCC = 0.958. The 95% confidence interval for accuracy was 94.7–97.6%. Accuracy was comparable between complete and draft assemblies, with 120 of 125 complete genomes (96.0%) and 475 of 492 draft assemblies (96.5%) correctly classified. Typing coverage was 100% in both groups; no assembly produced an untypeable result. All five errors among complete genomes were misclassifications between the closely related LOS A and B classes. Per-class performance was strong across all major classes: Class C achieved perfect recall (1.000), Class B achieved precision (0.971) and recall (0.982), and Class H achieved precision (0.971) and recall (0.985). Weighted F1-score across all classes was 0.964. The most common misclassification patterns were A → B (*n* = 5), B → A (*n* = 3), and A → R (*n* = 3). Per-class precision, recall, and F1-score are shown in Table 5, and the confusion matrix is shown in Figure 3.

**Table 4:** Overall performance metrics for LOS class typing (*n* = 617).

Metric	Value
Total assemblies	617
Correct	595
Incorrect	22
Accuracy	96.4%
Cohen’s κ	0.958
MCC	0.958
95% CI (accuracy)	0.947–0.976
Weighted F1-score	0.964
Balanced accuracy	0.894
Typing coverage	100%

**Table 5:** Per-class precision, recall, and F1-score for LOS class typing (classes with support ≥3; complete data in Supplementary Table S1).

LOS class	Precision	Recall	F1-score	Support
A	0.959	0.899	0.928	79
B	0.971	0.982	0.977	170
C	0.983	1.000	0.991	113
E	0.882	0.938	0.909	16
F	1.000	1.000	1.000	8
G	0.875	1.000	0.933	7
H	0.971	0.985	0.978	68
I	0.750	1.000	0.857	3
J	1.000	1.000	1.000	3
K	1.000	1.000	1.000	16
L	1.000	1.000	1.000	6
M	1.000	0.571	0.727	7
O	1.000	0.984	0.992	63
P	0.976	0.953	0.965	43
R	0.571	0.667	0.615	6
S	1.000	1.000	1.000	6

**Figure 3:**
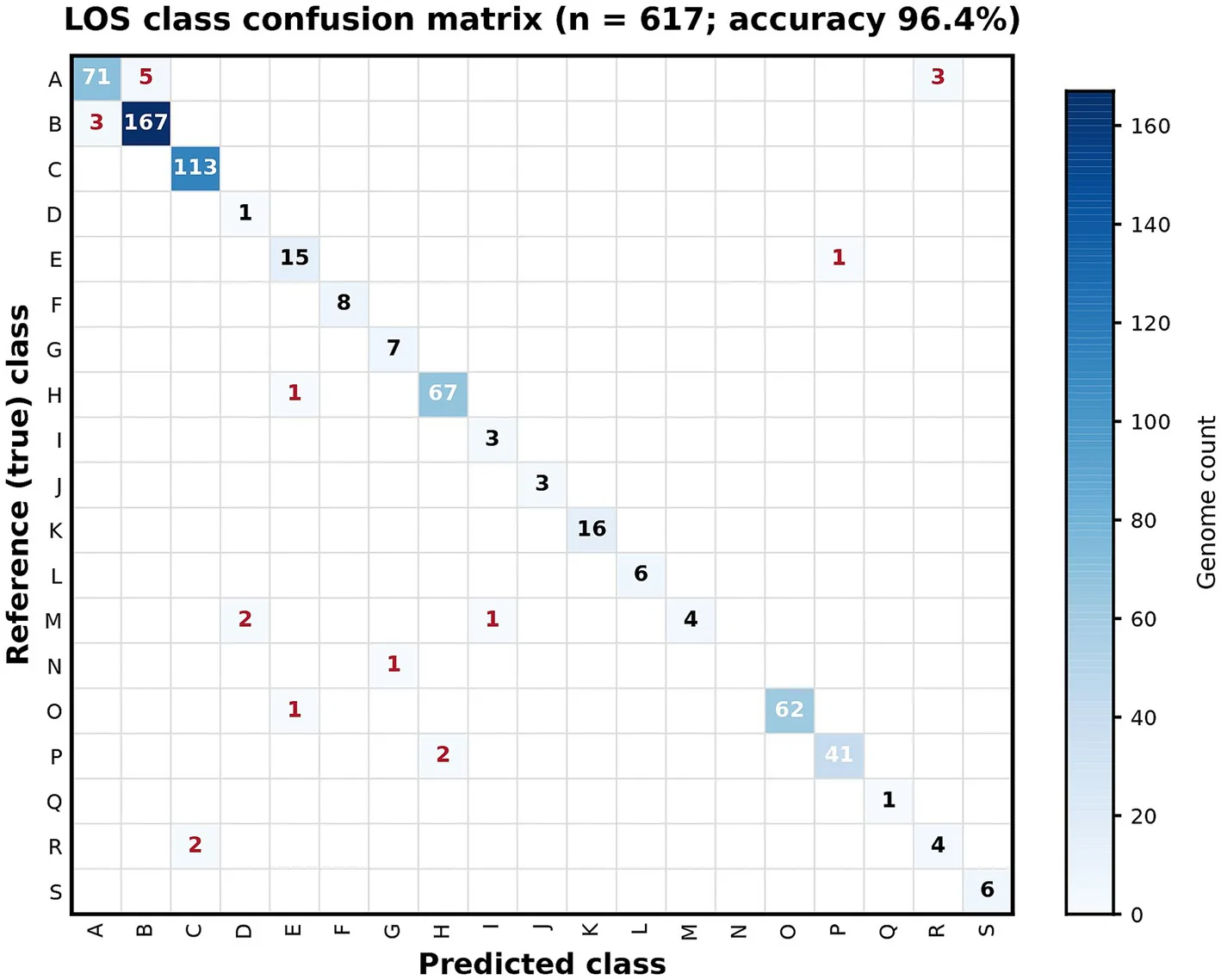
Confusion matrix for LOS class typing across 617 *C. jejuni* genome assemblies. Rows represent reference (true) LOS classes; columns represent CjejuniTyper-predicted classes. Diagonal cells show correct assignments; off-diagonal cells show misclassification counts.

### 3.3 Penner (HS) capsule validation

The retained Penner capsule type validation dataset comprised 96 *C. jejuni* genome assemblies covering 14 of the 31 CPS reference categories implemented in CjejuniTyper, collectively representing 27 individual Penner serotypes after expansion of complex-level calls. Several validated entries are complexes encompassing multiple serotypes: HS4c covers HS4, HS13, HS16, HS43, HS50, HS62, HS64, and HS65; HS5_31c covers HS5 and HS31; HS1c covers HS1 and HS44; and HS23_36c covers HS23 and HS36. Assemblies were retrieved from NCBI GenBank and PubMLST. HS19 (*n* = 35) and HS41 (*n* = 18) were the most represented types. Where NCBI or PubMLST metadata recorded individual serotype members rather than the complex designation used by CjejuniTyper, reference labels were manually harmonized to the complex level prior to evaluation (e.g., HS23 → HS23_36c and HS5 → HS5_31c). The remaining 17 implemented entries were not represented by assemblies with verified reference labels; their prediction accuracy remains to be formally established. The serotype distribution is shown in Table 6.

**Table 6:** Distribution of Penner (HS) types in the retained *C. jejuni* validation dataset (*n* = 96).

HS type	*n*
HS19	35
HS41	18
HS2	13
HS1c	7
HS4c	4
HS23_36c	3
HS55	3
HS5_31c	3
HS8_17c	3
HS9	2
HS6_7c	2
HS33_35	1
HS3	1
HS53	1

CjejuniTyper correctly assigned Penner type for 93 of 96 *C. jejuni* genome assemblies, yielding an overall accuracy of 96.9% (Table 7). Agreement with reference labels was high, with Cohen’s κ = 0.961 and MCC = 0.962. The 95% confidence interval for accuracy was 91.2–98.9%. Typing coverage was 100%. The pipeline achieved perfect recall for HS41 (18/18, recall 1.000) and HS2 (13/13, recall 1.000). HS19 recall was 0.971 (34/35). Three assemblies were misclassified: one HS19 predicted as HS1c, one HS4c as HS2, and one HS5_31c as HS15c. The HS4c complex therefore had one misclassification among four retained validation assemblies, corresponding to three-fourth correctly classified. Weighted F1-score was 0.973, and balanced accuracy was 0.956. Per-class metrics are shown in Table 8, and the confusion matrix is shown in Figure 4.

**Table 7:** Overall performance metrics for Penner (HS) capsule type prediction (*n* = 96).

Metric	Value
Total assemblies	96
Correct	93
Incorrect	3
Accuracy	96.9%
Cohen’s κ	0.961
MCC	0.962
95% CI (accuracy)	0.912–0.989
Weighted F1-score	0.973
Balanced accuracy	0.956
Typing coverage	100%

**Table 8:** Per-class precision, recall, and F1-score for Penner (HS) capsule type prediction.

HS type	Precision	Recall	F1-score	Support
HS1c	0.875	1.000	0.933	7
HS2	0.929	1.000	0.963	13
HS3	1.000	1.000	1.000	1
HS4c	1.000	0.750	0.857	4
HS5_31c	1.000	0.667	0.800	3
HS6_7c	1.000	1.000	1.000	2
HS8_17c	1.000	1.000	1.000	3
HS9	1.000	1.000	1.000	2
HS19	1.000	0.971	0.986	35
HS23_36c	1.000	1.000	1.000	3
HS33_35	1.000	1.000	1.000	1
HS41	1.000	1.000	1.000	18
HS53	1.000	1.000	1.000	1
HS55	1.000	1.000	1.000	3

**Figure 4:**
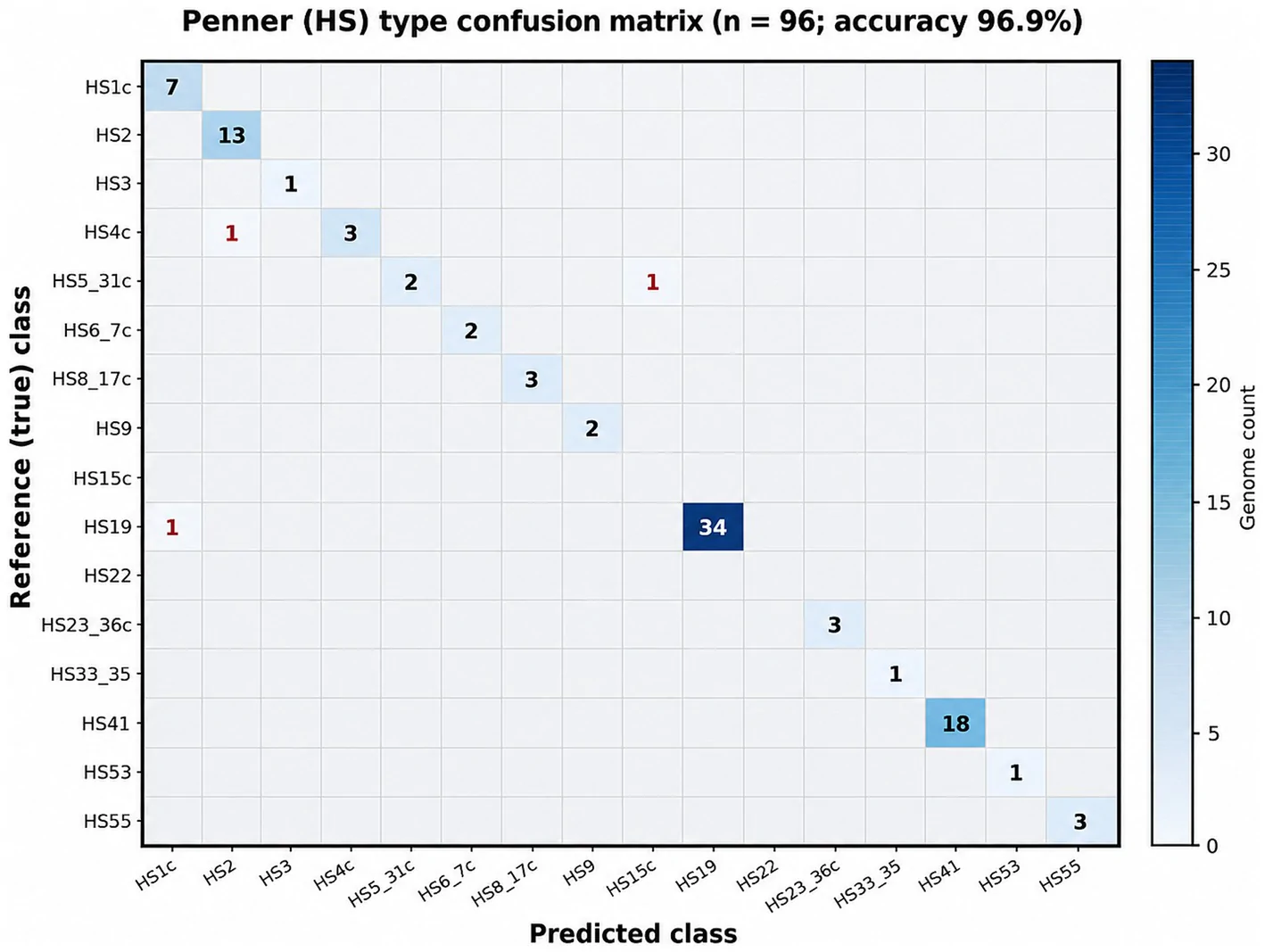
Confusion matrix for Penner (HS) capsule type prediction across 96 retained *C. jejuni* genome assemblies. Rows represent reference (true) serotypes; columns represent CjejuniTyper-predicted serotypes.

### 3.4 Validation studies: GBS-associated genomic profiling

Published LOS-class assignments were available for 84 of the 89 retained GBS-associated genomes. CjejuniTyper produced concordant assignments for 32 of 33 evaluable Bangladesh genomes (97.0%) and all 51 retained external genomes (100%), resulting in an overall LOS-class concordance of 83 of 84 genomes (98.8%). Five Bangladesh isolates with undetermined published LOS classes were excluded from this comparison (Table 9).

**Table 9:** Concordance between published and CjejuniTyper LOS-class assignments in GBS-associated genomes.

Cohort	Evaluable genomes	Concordant	Discordant	Concordance
Bangladesh GBS	33	32	1	97.0%
External GBS	51	51	0	100%
All GBS genomes	84	83	1	98.8%

For evaluation of the integrated GBS-associated genomic classification, HIGH and MODERATE results were considered positive profiles and LOW results negative. In the Bangladesh case–control cohort, positive profiles were identified in 34 of 38 GBS-associated isolates, while 14 of 24 enteritis controls had negative profiles. This corresponded to 89.5% sensitivity, 58.3% specificity, 77.4% accuracy, and 73.9% balanced accuracy. The association between a positive genomic profile and the GBS clinical group had an odds ratio of 11.90 (95% CI, 3.19–44.37; Fisher’s exact *p* = 0.000103) (Table 10). When the GBS-associated isolates were analysed without controls, positive profiles were detected in 34 of 38 Bangladesh isolates (89.5%; 95% Wilson CI, 75.9–95.8%), 47 of 51 retained external isolates (92.2%; 95% CI, 81.5–96.9%), and 81 of all 89 retained GBS-associated isolates (91.0%; 95% CI, 83.3–95.4%) (Table 11). In a secondary pooled analysis including all 89 retained GBS-associated genomes and 24 controls, 95 of 113 classifications were concordant with the reported cohort labels (Table 10).

**Table 10:** Performance of the integrated GBS-associated genomic classification.

Metric	Bangladesh case–control, *n* = 62	Pooled retained dataset, *n* = 113
True positives	34	81
False negatives	4	8
True negatives	14	14
False positives	10	10
Sensitivity	89.5%	91.0%
Specificity	58.3%	58.3%
Accuracy	77.4%	84.1%
Balanced accuracy	73.9%	74.7%
Positive predictive value	77.3%	89.0%
Negative predictive value	77.8%	63.6%
F1-score	82.9%	90.0%
Matthews correlation coefficient	0.513	0.510
Odds ratio	11.90	14.18
95% CI for odds ratio	3.19–44.37	4.77–42.12
Fisher’s exact *p*	0.000103	1.01 × 10^−6^

**Table 11:** Distribution of CjejuniTyper GBS-associated genomic profiles among GBS-associated isolates without controls.

Cohort	Total	High	Moderate	Low	High or moderate	Positive-profile detection rate
Bangladesh GBS	38	27	7	4	34	89.5%
External GBS	51	46	1	4	47	92.2%
All GBS-associated isolates	89	73	8	8	81	91.0%

Validation was constrained by the availability of publicly annotated reference genomes. While the LOS validation dataset included representatives of all implemented LOS classes, the Penner validation dataset contained verified reference labels for only 14 of the 31 implemented CPS types and complexes because suitable annotated genome assemblies are currently limited in public databases. Furthermore, CjejuniTyper currently supports the implemented reference LOS classes and CPS types included in its curated databases and does not encompass all known or potentially novel locus variants. Isolates carrying previously undescribed, highly divergent, or recombinant LOS or CPS loci may therefore be assigned to the closest available reference or remain unresolved. Future updates incorporating newly described reference sequences and expanded validation datasets will further improve the breadth and robustness of the pipeline. The GBS-associated evaluation was additionally limited by the absence of a matched control group for the 51 retained external genomes; these genomes were therefore used only to estimate the positive-profile detection rate. Because HIGH or MODERATE profiles were also identified in some enteritis controls, the integrated classification should be interpreted as genomic mimicry-associated profiling rather than patient-level clinical prediction.

## 4 Discussion

The results show that a BLAST-based genome analysis method provides a reliable way to determine *C. jejuni* CPS type and LOS class using a genome-wide characterization of whole genomes, yielding total accuracy levels of 96.4 and 96.9% for LOS and Penner types, respectively. Both LOS and Penner types had very high agreement statistics (κ ≥ 0.958; MCC ≥ 0.958). There were no instances of incomplete typing coverage in the retained validation datasets. Thus, the study supports that a completely automated *in silico* workflow could serve as an alternative to traditional culture-dependent Penner serotyping and LOS classification. In the additional GBS-associated cohort analysis, CjejuniTyper showed 98.8% concordance with published LOS classes and identified HIGH or MODERATE genomic profiles in 81 of 89 retained GBS-associated genomes (91.0%).

To our knowledge, CjejuniTyper represents the first pipeline that includes all five of the previously identified distinct processes: Penner CPS-type identification, LOS assignment, *Cst-II* allele determination, ganglioside-mimic prediction, and an integrated GBS risk assessment, utilizing a single workflow operating directly on genome assemblies.

Prior molecular typing methodologies for *C. jejuni* have focused solely on CPS or LOS classification separately. Expanded multiplex PCR methods have been developed to include all 47 identified Penner serotypes by targeting variable CPS loci (Poly et al., 2011; Poly et al., 2015), and several studies have identified PCR-based methodologies for detecting LOS classes (Parker et al., 2008; Hameed et al., 2022). Because these laboratory methods generally require separate assays, these approaches do not integrate *Cst-II* allele status, ganglioside-mimic prediction, or GBS risk scoring. While *in silico* PCR has been employed to analyse genome-sequence data to identify CPS types (Khemnu et al., 2023), this application only assessed capsule-type and did not extend to LOS-classification or GBS risk predictions. Although MLST identifies clonal lineage groups associated with GBS risk, it does not assess the structure of surface antigens directly (Dingle et al., 2001). CjejuniTyper fills the gap left by prior applications in identifying all of these analytical components in one operation and produces a comprehensive output linking typing characteristics with clinical indicators of risk without requiring further laboratory processing.

Classes A and B accounted for eight of the 22 LOS misclassifications (36%), including five A → B and three B → A errors. The regions responsible for distinguishing among members of these two classes are highly conserved across their respective sialylation-cassettes; however, the primary distinctions between them lie in the length and gene composition of the region distal to the core biosynthesis genes; therefore, when genomic data exist as partial or fragmentary assemblies of these regions (as occurs in draft assemblies), the differences in alignment lengths employed to discern A vs. B calls may be inadequate to produce definitive assignments. Of particular interest regarding this issue is that all five A/B errors found in complete-genome sequences occur in strains located structurally at the boundaries of these two classifications. It is possible that some or all of these represent actual ambiguities in the classification whether due to inaccuracies within the CjejuniTyper algorithm or those resulting from ambiguous reference labels produced by Hameed et al., employing a Megablast-based methodology that may prioritize differing scores for borderline A/B loci (Hameed et al., 2022). This example illustrates a fundamental problem in LOS classification: although A and B are the two most clinically relevant sialylated classes, they are also the two most difficult to distinguish via computational means. Independent validation against phenotypically confirmed reference strains would provide clarity regarding the utility of CjejuniTyper’s LOS classification capabilities.

Because LOS Classes A and B are highly similar, CjejuniTyper currently reports the single best-supported class rather than an A/B ambiguity category. Although this boundary accounted for several misclassifications, overall LOS accuracy remained high, and introducing a broad A/B mixed call could reduce specificity by flagging many otherwise correctly classified isolates. Future development will therefore focus on identifying additional discriminatory sequence features or complementary approaches beyond whole-locus BLAST similarity before implementing a dedicated ambiguity flag for these closely related classes.

Three Class A assemblies were identified as Class R. Class R is a structural mosaic in which the *orf11* gene of the Class A locus has been replaced by an orf16-like gene from Class D or F; the remainder of the locus is almost indistinguishable from Class A; thus, the presence or absence of *orf11* serves as the primary characteristic to distinguish the two (Parker et al., 2008). The *orf11*-based disambiguation method in CjejuniTyper resolves this ambiguity at high-identity STRICT levels; however, in draft assemblies where *orf11* is fragmented or does not reach the minimum detection threshold, its apparent absence can result in a false Class R call, causing a true Class A locus to be designated as Class R. It was impossible to determine whether these three assemblies are actual Class A strains with an undetectable *orf11* or possess a different locus using sequence data. Three assemblies classified as Class M were incorrectly assigned, with two being designated as Class D and one as Class I. Class M represents a mosaic of Class A and Class D; when the A-origin part of a fragmented assembly is incomplete, the D-origin part of the assembly may contribute disproportionately to the overall alignment score, thereby causing a true Class M locus to be designated as Class D; conversely, if the residual signal aligns best with another class, then it is designated as such (Parker et al., 2008). Two Class R assemblies were designated as Class C, and two Class P assemblies were designated as Class H; both pairs share structural similarities within the tier thresholds employed and in both cases the misclassified assemblies exhibited elevated contig counts indicative of fragmented loci. The remaining four incorrect classifications (H → E, N → G, O → E, and E → P) each occurred once in classes which represent only a small fraction of assemblies included in the validation set (support 1–16); due to a solitary borderline assembly in each of these underrepresented classes, there exists uncertainty regarding whether errors observed resulted from limitations in pipeline processes or inaccuracies in metadata. Since publicly accessible LOS class annotations are not always confirmed by experimental evidence, and since they often depend upon computational predictions, these ambiguities remain unresolved.

Each of the three retained Penner type classification errors involves serotype complexes that exhibit known sequence homology. The first error involves a draft HS19 assembly that was classified as HS1/44c; the second involves a draft HS4c assembly that was classified as HS2; and the third involves a draft HS5_31c assembly that was classified as HS15c. In each instance, the misclassified assembly was a draft genome with a fragmented CPS locus, and the marker gene required for the correct designation was either absent from the assembly or fell below the Tier A identity threshold required for accurate designation. Similar to LOS, metadata inaccuracy cannot be excluded as a potential contributor to these classification errors; CPS-type designations present in public databases are generally based on serologic assays performed many years prior to current research efforts. Previous studies have demonstrated discrepancies in annotation designations across databases (Pike et al., 2013; Poly et al., 2015). Therefore, due to the relatively large number of HS19 and HS41 strains present in the validation set, reflecting their clinical prominence, caution should be exercised when interpreting performance estimates for less represented serotypes, especially those that are represented by only 1–3 assemblies.

It is essential to acknowledge several limitations associated with the validation strategy. The LOS validation dataset exhibits significant imbalance in terms of class distribution; Classes B (*n* = 170), C (*n* = 113), and A (*n* = 79) comprise approximately 59% of all assemblies in this dataset, whereas Classes D, N, and Q are each represented by a single genome. Thus, performance estimates generated for rare classes are subject to statistical unreliability, and additional annotated genomes for these classes will be necessary before definitive conclusions can be made regarding per-class accuracy. Analogously, the Penner validation set of 96 *C. jejuni* assemblies includes representatives from 14 of the 31 CPS reference entries implemented in CjejuniTyper, collectively representing 27 distinct Penner serotypes because certain validated entries include complexes encompassing numerous serotypes (for example, HS4c contains HS4, HS13, HS16, HS43, HS50, HS62, HS64, and HS65; similarly, HS5_31c corresponds to both HS5 and HS31). The remaining 17 implemented types/complexes were not represented by assemblies with verified reference labels at the time of validation; thus, their predictive accuracy must still be formally established. Consequently, interpretation of CPS typing accuracy must occur with consideration of these caveats.

A further limitation of the Penner module is its marker-primary design. Once a serotype-specific marker gene is detected, its complex-level assignment will take precedence over the full-locus best hit, except for a small set of curated cross-reactive pairs. There are some very rare instances in which this hierarchy can suppress an otherwise robust full-locus match; for example, a strain of HS31 has an HS15-like marker gene and may therefore be reported as HS15c at the complex level, although its entire CPS locus is most similar to HS5_31c. Such cases should be interpreted cautiously, especially when marker-based and full-locus evidence is discordant. Manual inspection of the intermediate HS BLAST results is recommended for cross-reactive complexes or unexpected calls. Since well-documented, serologically confirmed Penner genomes remain very scarce and are generally based on typing performed using decades-old antisera, both the current marker versus locus precedence and its conflict-pair exceptions are necessarily temporary. As more well-documented genomes become available, this precedence should be formally revisited and, for certain cross-reactive pairs, a locus-primary decision rule may provide better accuracy than the current marker-primary default.

The evaluation in the Bangladesh case–control cohort showed that the integrated classification was significantly associated with the reported clinical group. HIGH or MODERATE profiles were detected in 34 of 38 GBS-associated isolates, corresponding to a sensitivity of 89.5%. The odds of receiving a positive genomic profile were 11.9-fold higher among GBS-associated isolates than among enteritis controls. The retained external dataset provided a similar result, with 47 of 51 GBS-associated genomes receiving HIGH or MODERATE profiles. Across both studies, 81 of 89 retained GBS-associated genomes were positive, giving an overall positive-profile detection rate of 91.0%. These findings indicate that the rules capture the canonical genomic characteristics found in most GBS-associated *C. jejuni* isolates across geographically diverse collections (Nayeem et al., 2025; Hayat et al., 2026).

The lower specificity observed in the Bangladesh case–control analysis was mainly caused by 10 enteritis controls that received HIGH or MODERATE classifications. Importantly, these controls should not be interpreted simply as LOS typing errors. Isolate-level published LOS classifications were not available for the controls in the metadata used in this evaluation; however, CjejuniTyper assigned all 10 positive controls to sialylated LOS classes, comprising four Class A and six Class B isolates. Because Classes A and B contain the canonical sialylation machinery associated with ganglioside mimicry, their assignment produces a positive LOS-based GBS signal and strongly contributes to the integrated HIGH or MODERATE classification. So, these 10 isolates are false positives relative to their enteritis cohort labels, but they are not necessarily analytical false positives. Rather, the program detected the genomic features that it was designed to identify. The high LOS typing concordance observed in the validation dataset and the 98.8% concordance among GBS-associated genomes further support the plausibility of these LOS assignments, although experimental confirmation of the control LOS structures was not available.

Targeted analysis of the 10 positive controls also showed that they were not genetically uniform. Protein-coding sequences were predicted with Prodigal and screened by BLASTP against curated LOS-pathway protein references. Four of the 10 positive controls contained full-length, high-identity *Cst-II*, *CgtA, CgtB, NeuB,* and *NeuC* proteins, supporting the presence of a complete intact-looking mimicry-associated pathway rather than detection based only on partial nucleotide BLAST matches. Of the remaining six, one contained intact *neuB* and *neuC* but lacked an intact sialyltransferase and the accessory transferases, two contained *Cst-II*, *neuB,* and *neuC* but lacked complete *cgtA* and *cgtB*, and three contained only partial *cgtA* matches of approximately 63% coverage without a complete *cgtB*. No long homopolymeric G/C tract was identified in the targeted genes of these controls. These findings explain why some controls received HIGH profiles while others received MODERATE profiles and demonstrate different levels of mimicry-associated genetic potential. They do not prove that the genes were expressed or that the corresponding ganglioside-like LOS structures were produced. Biological overlap between GBS-associated and enteritis isolates is also consistent with the source study, which detected *Cst-II* in 9 of 24 enteritis controls and did not identify a single LOS gene or region that was exclusive to the GBS-associated group (Nayeem et al., 2025).

The eight GBS-associated isolates receiving LOW profiles also require biological rather than purely statistical interpretation. Four external isolates had published LOS Classes D, F, H, and P, and CjejuniTyper assigned the same respective classes in every case. These were therefore correct LOS calls, but the isolates received LOW profiles because D, F, H, and P are not included among the canonical sialylated LOS Classes A, B, C, M, and R used by the current scoring framework. In the Bangladesh dataset, BD94 had a published Class W assignment but was predicted as Class H because a suitable validated Class W reference was not incorporated into the current CjejuniTyper LOS reference database. Nevertheless, neither Class W nor Class H is included among the canonical sialylated classes, so this LOS mismatch did not change the resulting LOW genomic profile. The other three LOW Bangladesh isolates had undetermined published LOS assignments; two remained untypeable, and one was assigned Class F.

The presence of GBS-associated isolates with Classes D, F, H, P, or W does not demonstrate that these classes are established ganglioside-mimic LOS classes, nor does it mean that the patient diagnoses were incorrect. GBS development depends on the interaction of bacterial, immunological, and host factors, and the bacterial isolate associated with a patient may lack the canonical genomic pathway detected by CjejuniTyper. These uncommon observations may represent non-canonical disease mechanisms, host-dependent immune responses, incomplete causal attribution of the preceding infection, or rare LOS pathways that have not yet been sufficiently characterized. Current evidence is therefore insufficient to modify the scoring logic to classify D, F, H, P, or W as positive GBS-associated LOS classes. The rules should be reconsidered when larger clinically characterized datasets, functional LOS analyses, and experimental evidence become available.

Tables 10, 11 consequently answer different questions. The case–control analysis in Table 10 evaluates association with the reported clinical groups and demonstrates high sensitivity but moderate specificity. Table 11 evaluates only how frequently the canonical genomic profile was detected among isolates already reported from GBS patients and shows a detection rate of 91.0%. The control isolates should not be excluded from the primary analysis because they reveal the central biological limitation of the classification: sialylated LOS and mimicry-associated genes can occur in enteritis controls without a diagnosis of GBS. At the same time, the high GBS-only detection rate shows that the current rules identify the canonical genomic profile in most GBS-associated isolates. CjejuniTyper should therefore be interpreted as a genomic typing and mimicry-potential screening tool rather than a standalone predictor of whether an infected individual will develop GBS.

Currently, CjejuniTyper uses 19 LOS classes (A–S) as defined by Parker et al. (2008) and extended by Hameed et al. (2022). Classes T, U, V, and W were not included in the current curated database because suitable validated references were not incorporated in this version. Genomes containing these classes would either be placed in the closest implemented class or designated as untypeable. More recently discovered LOS classes—including the CDC11–CDC19 classes found in Chinese isolates and additional new loci in clinical collections from South and Southeast Asia—are not yet included (Khemnu et al., 2023; Wang et al., 2022). As these new classes are further studied and reference sequences become available, they can be added to the LOS database. Similarly, CPS typing uses 35 non-duplicate CPS reference labels that collectively represent the 47 classical Penner HS serotypes at the capsule-complex level, while CjejuniTyper reports these as 31 conservative HS categories/complexes. Newly identified capsular loci described by Khemnu et al. (2023) are not currently represented in the database and would therefore produce untypeable results. The reference databases will also be expanded as well-characterized genomes representing rare LOS and Penner classes become available. Some rare classes remain difficult to validate because suitable reference assemblies with reliable typing metadata are scarce. Although assembly fragmentation can affect classification when discriminatory regions are interrupted or absent, it was not associated with reduced overall LOS performance in the present dataset: accuracy was 96.0% for complete genomes and 96.5% for draft assemblies, with 100% typeability in both groups. Several errors therefore appear to reflect intrinsic sequence similarity between closely related loci rather than assembly fragmentation alone. Further development will focus on improving database coverage, developing long-read assembly support, and investigating web-based interfaces to increase accessibility for laboratories lacking local computational resources.

CjejuniTyper is intended to perform scalable genomic analysis on a range of assemblies, from individual assemblies to large-scale surveillance collections. The modular architecture allows for batch processing of multiple assemblies, simultaneous execution via user-specified job distribution, and incorporation with standard Linux-based bioinformatics workflows. These capabilities facilitate deployment in research laboratories and public health laboratory environments where large volumes of whole-genome sequencing data are being processed. In conclusion, CjejuniTyper offers a scalable, reproducible, fully automated alternative to traditional Penner serotyping and LOS class determination for *C. jejuni* whole-genome assemblies and incorporates multiple typing schemes and GBS risk indicators into a single output file. The pipeline is ideally suited to high-throughput genomic surveillance environments where numerous assemblies need to be typed rapidly and inexpensively without employing time-consuming and expensive culture-based serology methods. The methodology and results described here demonstrate that marker-gene BLAST combined with full-locus alignment and a rule-based interpretation engine can achieve high concordance with curated reference classifications, supporting the ongoing transition from phenotypic to genomic characterization of *C. jejuni* in clinical and public health microbiology.

## Data Availability

The datasets presented in this study can be found in online repositories. The names of the repository/repositories and accession number(s) can be found in the article/Supplementary material.

## Footnotes

1
https://github.com/Gagi1993/CjejuniTyper
